# Nuclear magnetic resonance-based lipid metabolite profiles for differentiation of patients with liver cirrhosis with and without hepatocellular carcinoma

**DOI:** 10.1007/s00432-025-06178-x

**Published:** 2025-04-04

**Authors:** Luigi Nardone, Marianna Alunni-Fabbroni, Regina Schinner, Sabine Weber, Julia Mayerle, Eric Schiffer, Sebastian de Jel, Max Seidensticker, Peter Malfertheiner, Jens Ricke

**Affiliations:** 1 Department of Medicine, Institute of Radiology, University of Udine, Piazzale Santa Maria Della Misericordia 15, 33100 Udine, Italy; 2 Department of Radiology, University Hospital, LMU Munich, Marchioninistrasse 15, 81377 Munich, Germany; 3 Department of Medicine II, University Hospital, LMU Munich, Marchioninistrasse 15, 81377 Munich, Germany; 4 Numares AG, Am BioPark 9, 93053 Regensburg, Germany

**Keywords:** Metabolomics, NMR, Lipid profiles, Early diagnosis

## Abstract

**Background:**

Hepatocellular carcinoma is frequently unrecognized in its early stage limiting the access to the first therapeutic steps resulting in a low cure rate. Therefore, an early diagnosis is crucial. In this scenario the analysis of lipidome and metabolome emerged as a promising tool for early detection.

**Aims:**

Aim of the study was to characterize metabolomic profiles as novel markers of early hepatocellular carcinoma.

**Methods:**

Serum basal levels of metabolites, isolated from a cohort of 90 patients (n = 30 early stage; n = 30 advanced stage; n = 30 liver cirrhosis) were analysed using a nuclear magnetic resonance spectroscopy platform. To assess the predictive value of nuclear magnetic resonance profiles, we included the magnetic resonance imaging follow up of control patients with liver cirrhosis.

**Results:**

Significant differences were observed in the levels of individual parameters that included total cholesterol, LDL and HDL subclasses, Isoleucine, Valine, Triglycerides, Lactate, Alanine, Albumin, alpha Fetoprotein, Dimethylamine, Glycerol, and total Bilirubin levels in cancer compared to liver cirrhosis (*p* < 0.05). Furthermore, a significant difference in glycerol levels (p < 0.05) and a decreasing trend in dimethylamine were observed in cirrhotic patients who later developed HCC (16%, n = 5). Retrospective MRI analysis revealed precursor lesions in 3/5 patients, initially not classified as HCC due to their size and hemodynamic features.

**Conclusion:**

Nuclear magnetic resonance based assessment of lipidomic and metabolomic profiles permit the differentiation of cancer from liver cirrhosis. The data obtained suggests a possible role of lipidomic based serum profiles for early detection.

**Supplementary Information:**

The online version contains supplementary material available at 10.1007/s00432-025-06178-x.

## Introduction

Hepatocellular carcinoma (HCC) is one of most frequent cancers worldwide, reaching about 1 million new cases per year, and third for mortality rate according to the global cancer observatory (GLOBOCAN) (Sung et al. 2021). The evolving epidemiological data reveals a notable transition in the underlying risk factors for HCC, shifting from predominantly virus-related to non-viral liver diseases, e.g. metabolic associated fatty liver disease MASLD (Crane et al. 2023). Early diagnosis of HCC leads to approximately a 60% cure rate compared to a cure rate of 15% in advanced HCC (Wu et al. 2020). However, HCC is frequently unrecognized in its early stage, leading to a late diagnosis and therefore a low cure rate that typically includes a 5-year survival rate less than 15% (Wu et al. 2020). Several methods and strategies are currently applied for early detection of HCC, including blood biomarkers and imaging. Patients with liver cirrhosis (LC) receive surveillance at regular intervals with ultrasound, however lesions smaller than 10 mm are missed in about 30% of cases (Llovet et al. 2021; Sparchez et al. 2021). Alpha-fetoprotein (AFP), the most extensively evaluated biomarker, shows a limitation in sensitivity being less than 70% (Piñero et al. 2020; Johnson et al. 2022). Studies on tumor metabolism have unveiled new potential biomarkers, including gene expression assays (Okabe et al. 2001), liquid biopsy (Stechele et al. 2023; Hirner-Eppeneder et al. 2023; Alunni-Fabbroni et al. 2021; Alunni-Fabbroni et al. 2019), and systemic markers of inflammation (Öcal et al. 2022; Kästle et al. 2023). In recent years, the analysis of lipidome and metabolome emerged as a promising novel tool for HCC early detection. Elevated levels of both lipogenesis and lipolysis, in fact, contribute to the growth, proliferation, and survival of cancer cells (Wang and Han 2016). Changes in lipidomics and metabolomics were reported to allow early diagnosis of HCC and predict outcomes (Caponigro et al. 2023; Wu et al. 2023; Rashid et al. 2023). Nuclear magnetic resonance (NMR) spectroscopy is an analytical method for metabolome analysis, characterized by low invasiveness, simple sample processing, and high analytical reproducibility. NMR-based assessment of lipidomic and metabolomic profiles in predicting overall survival and early diagnosis has been reported before by our group and others (Geyer et al. 2021; Gao et al. 2009; Liu et al. 2014). The novelty of this study was to analyse different NMR profiles in patients with LC with different stages of HCC and in patients with LC without HCC, both with liver insufficiency graded according to the Child–Pugh Score (CPS), to identify novel markers of early HCC. Our study could significantly contribute to the existing literature, not only through the analysis of NMR profiles but also by incorporating subsequent radiological MRI follow-up. This integrated approach allows for the confirmation and more precise selection of identified biomarkers. By combining radiological data with biological data, we gain a more comprehensive and accurate understanding of the patient's health status, thereby enhancing the ability to diagnose and monitor HCC.

## Material and methods

### Study design

The present study is a sub-study of the prospective, randomized-controlled, multi-center phase II SORAMIC trial (EudraCT 2009–012576-27, NCT01126645), which was conducted in 12 countries in Europe and Turkey (Ricke et al. 2019, 2015), in combination with a control group. The study was approved by the institutional review boards and conducted according to the ethical principles expressed in the Declaration of Helsinki. Written informed consent was obtained from all participants. In total, 90 patients were included in this study**,** of which 30 patients from the SORAMIC cohort (recruited between February 2011 and January 2016) presented with early HCC (BCLC A), liver cirrhosis (LC) and a CPS between A and C; 30 patients from the SORAMIC cohort (recruited between February 2011 and August 2015) presented with advanced HCC (BCLC B or C), LC and a CPS between A and C; 30 patients (recruited between May 2019 and June 2019) presented with LC without HCC and a CPS between A and C.

Peripheral blood (5 mL) was drawn in S-Monovette serum tubes (Sarstedt AG, Nümbrecht, Germany) and immediately processed (centrifugation 3000 rpm, 5 min, 4 °C) to collect serum, which was aliquoted and stored at − 80° C until use.

### NMR AXINON^®^ platform

Targeted metabolomic analysis was performed using the standardized NMR AXINON^®^ platform (Numares AG, Regensburg, Germany). Serum was thawed at room temperature and prepared for NMR analysis. To this end, 630 µL serum of each sample was mixed with 70 µL of the AXINON^®^ serum additive solution and a total of 600 µL was transferred to 5 mm NMR tubes. If necessary, multiple aliquots of the same serum sample were pooled to reach the required minimum sample volume. The samples were run in several batches, each including calibration and two process control samples and were kept at 6 °C in the SampleJet auto-feeder until measurement on a Bruker Avance III 600 MHz spectrometer equipped with a 5 mm PATXI probe and automatic Z gradients shimming (Bruker Corporation, Billerica, MA, USA). The NMR measurement was carried out at 37 °C after pre-heating the samples at 37 °C for 7.5 min. The acquisition and processing of NMR spectra and the quantification of NMR signals for a lipoprotein subclass and a small metabolite analysis were carried out as described (Stratmann et al. 2016; Ehrich et al. 2021). All tested parameters are displayed in Table 1.

**Table 1:** Metabolite and lipid parameters that were analyzed in the present study using the standardized AXINON^®^ platform (Numares AG, Regensburg, Germany)

Parameter Description	Name	Unit
Creatine	Concentration of serum creatine	µmol/L
Creatinine	Concentration of serum creatinine	µmol/L
Dimethylamine	Concentration of serum dimethylamine	µmol/L
Dimethylsulfone	Concentration of serum dimethyl sulfone	µmol/L
Glycerol	Concentration of serum glycerol	µmol/L
Isoleucine	Concentration of serum isoleucine	µmol/L
Myo-Inositol	Concentration of serum myo-inositol	µmol/L
Valine	Concentration of serum valine	µmol/L
GFR(NMR)	Glomerular filtration rate estimated from metabolite constellation	mL/min/1.73 m^2^
LVLDL-P	Concentration of large VLDL particles	nmol/L
LDL-P	Concentration of LDL particles	nmol/L
LLDL-P	Concentration of large LDL particles	nmol/L
SLDL-P	Concentration of small LDL particles	nmol/L
HDL-P	Concentration of HDL particles	nmol/L
LHDL-P	Concentration of large HDL particles	nmol/L
SHDL-P	Concentration of small HDL particles	nmol/L
VLDL-s	Mean diameter of VLDL particles	nm
LDL-s	Mean diameter of LDL particles	nm
HDL-s	Mean diameter of HDL particles	nm
VLDL-c	Cholesterol concentration in VLDL class	mg/dL
IDL-c	Cholesterol concentration in IDL class	mg/dL
LDL.A-c	Cholesterol concentration in LDL subclass A (large particles)	mg/dL
LDL.B-c	Cholesterol concentration in LDL subclass B (medium-sized particles)	mg/dL
LDL.C–c	Cholesterol concentration in LDL subclass C (small particles)	mg/dL
HDL.A-c	Cholesterol concentration in HDL subclass A (large particles)	mg/dL
HDL.B-c	Cholesterol concentration in HDL subclass B (medium-sized particles)	mg/dL
HDL.C–c	Cholesterol concentration in HDL subclass C (small particles)	mg/dL
Total-Cholesterol	Concentration of total cholesterol in serum	mg/dL
LDL-Cholesterol	Concentration of LDL-cholesterol in serum	mg/dL
HDL-Cholesterol	Concentration of HDL-cholesterol in serum	mg/dL
Triglycerides	Concentration of total triglycerides in serum	mg/dL
Alanine	Concentration of alanine in serum	µmol/L
Leucine	Concentration of leucine in serum	µmol/L

### Statistical analysis

All statistical analyses were performed using SAS Version 9.4 for Windows (Copyright SAS Institute Inc., Cary, NC, USA), R statistical software (version 4.1.1), and SPSS IBM (version 35.0.0.0). Serum metabolite concentrations among different subgroups were compared using ANOVA, followed by post hoc analysis with Tukey’s test. ANOVA was also used to assess differences based on sex. A false discovery rate (FDR) correction with adjusted p values was applied to account for multiple comparisons. Binary logistic regression was performed to evaluate whether metabolite alterations were independent of liver function (Child–Pugh score) and HCC stage. Linear regression analysis was conducted to assess the correlation between albumin, total bilirubin, and HCC stage, adjusting for liver function. Correlations were evaluated using Chi-square and Fisher’s exact tests. A t test was used for pairwise comparisons, and statistical significance was set at α ≤ 0.05.

## Results

### Patients’ clinical characteristics

A total of 90 patients with a mean age of 62 years (range 26–83 years) with a male predominance (73 men, 17 women; ratio 4.3:1) was included in this study. The three subgroups consisted of patients with early HCC as defined by BCLC stage 0 or A (n = 30), advanced HCC as defined by BCLC stage B – C (n = 30), and LC (n = 30). Early HCC patients were 24 male and 6 female (mean age 67 years, range 53–83 years), while advanced HCC patients were 28 male and 2 female (mean age 66 years, range 41–79 years) and LC patients were 21 male and 9 female (mean age 53 years, range 30–81 years). All patients presented different grades of liver failure, classified with a CPS between A (n = 62) and B/C (n = 28). The predominant etiologies of cirrhosis were alcohol (n = 47) and viral infection (HBV positive n = 7, HCV positive n = 17). The patients’ characteristics are given in Table 2.

**Table 2:** Patients’ characteristics

Variables	Total	Liver Cirrhosis	Early HCC	Advanced HCC	*p* value
	(n = 90)	(n = 30)	(n = 30)	(n = 30)	
Gender					0.085
Male	73	21	28	24	
Female	17	9	2	6	
Age (years)					< 0.0001
Mean (SD)	62.0 (11.5)	53.0 (12.3)	67.0 (8.2)	65.9 (8.4)	
Median	64	52	65.5	67.5	
Min–Max	30–83	30–81	53–83	41–79	
Age (years)					0.026
> 65	37	15	11	11	
≤ 65	53	15	19	19	
Child–Pugh					0.0005
A	62	13	25	24	
B/C	28	17	5	6	
Etiology					0.2934
Alcohol	47	17	18	12	
NASH	2	2	0	0	
AIH	1	1	0	0	
PCS	2	2	0	0	
HBV	7	2	1	4	
HCV	17	3	7	7	
Medication	1	1	0	0	
Other	13	2	4	7	
BMI					
Mean (SD)	27.1 (4.3)	25.5 (4.1)	27.7 (3.2)	28.1 (4.8)	
Median	26.3	25.3	27.1	27.3	
Min–Max	18.2–38	18.2–35.5	22.8–35.3	19.5–38	
**BMI**					0.096
Normal	30	15	7	8	
Overweight	43	11	16	14	
Obese	20	5	7	8	
**AFP** (ng/mL)					0.0001
> 10	34	3	10	21	
≤ 10	51	26	19	6	
N.a	5	1	1	3	

Baseline characteristics of all included patients with liver cirrhosis (n = 30), early HCC (n = 30) and advanced HCC (n = 30). Fischer’s exact test and chi-square test were used for the statical analysis

*BMI* body mass index, *HBV* hepatitis B virus, *HCC* hepatocellular carcinoma, *HCV* hepatitis C virus, *NASH* non-alcoholic steatohepatitis, *AIH* autoimmune hepatitis, *PCS* primary sclerosing cholangitis, *SD* standard deviation

Due to the gender imbalance with a clear male predominance, we performed an ANOVA to assess whether there were statistically significant differences in each metabolite between sexes. Only LDL_p (p value = 0.05), HDL_s (p value = 0.03), LDL_B_c (p value = 0.02), lactate (p value < 0.01), and total bilirubin (p value < 0.01) demonstrated statistically significant results.

### Lipids’ profile

The NMR-based metabolomic panel used in this study includes metabolites involved in liver function, amino acid metabolism, and lipid transport. These pathways are highly relevant to hepatic disease progression, as metabolic shifts occur during the transition from cirrhosis to HCC. Several components of this panel, such as creatinine, glycerol, dimethylamine, and lipid subclasses, have been previously associated with liver dysfunction and oncogenesis. The panel’s structure allows for a broad yet targeted assessment of metabolic alterations, making it a valuable tool for identifying potential predictive markers of early HCC.

**Fig. 1:**
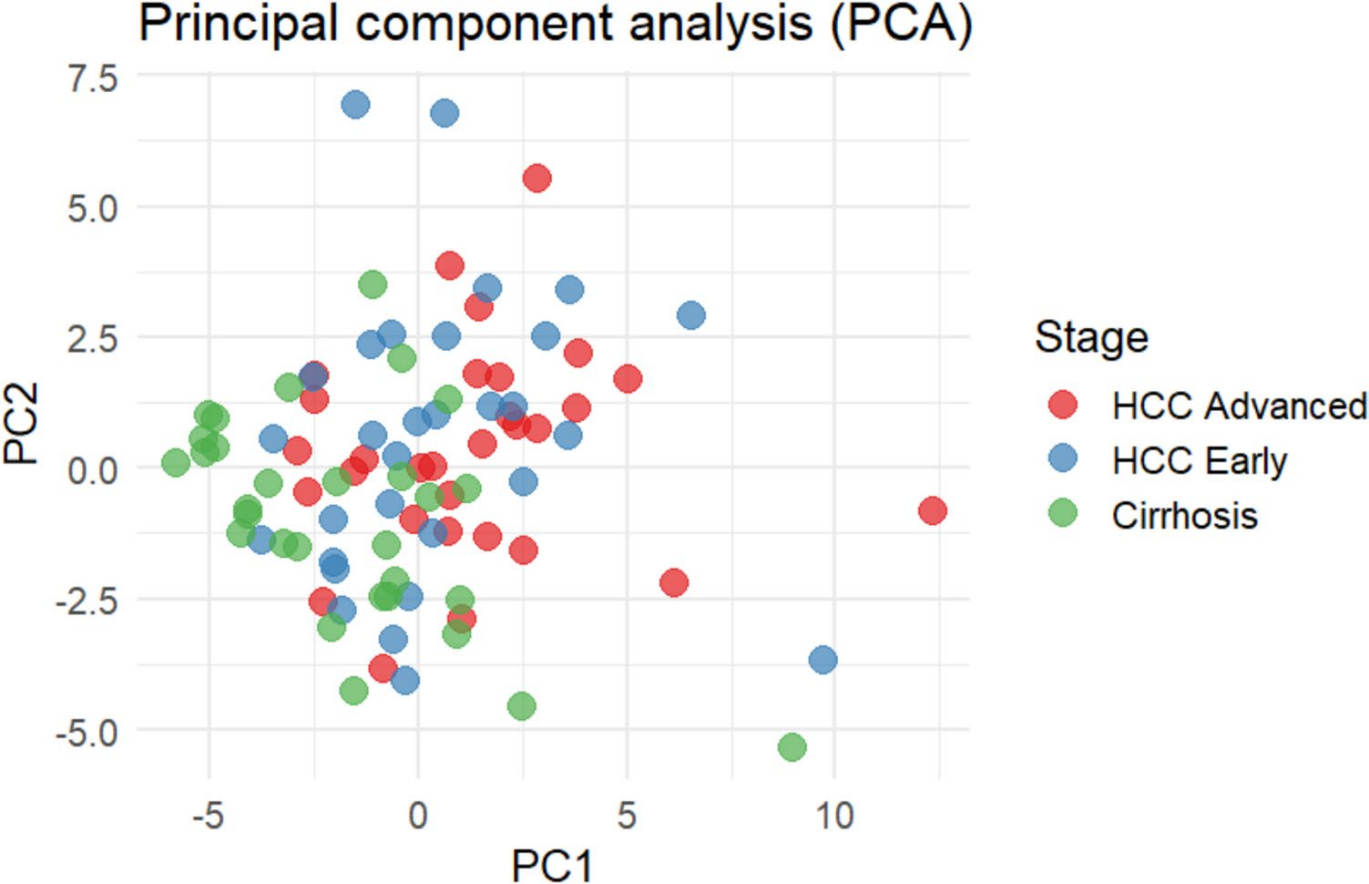
Principal component analysis (PCA) performed on standardized data. The figure shows a plot of the first two principal components, with points color-coded according to the HCC group. PCA was used to reduce the data's dimensionality and visualize the differences between groups, highlighting potential distinct patterns associated with the HCC group

**Fig. 2:**
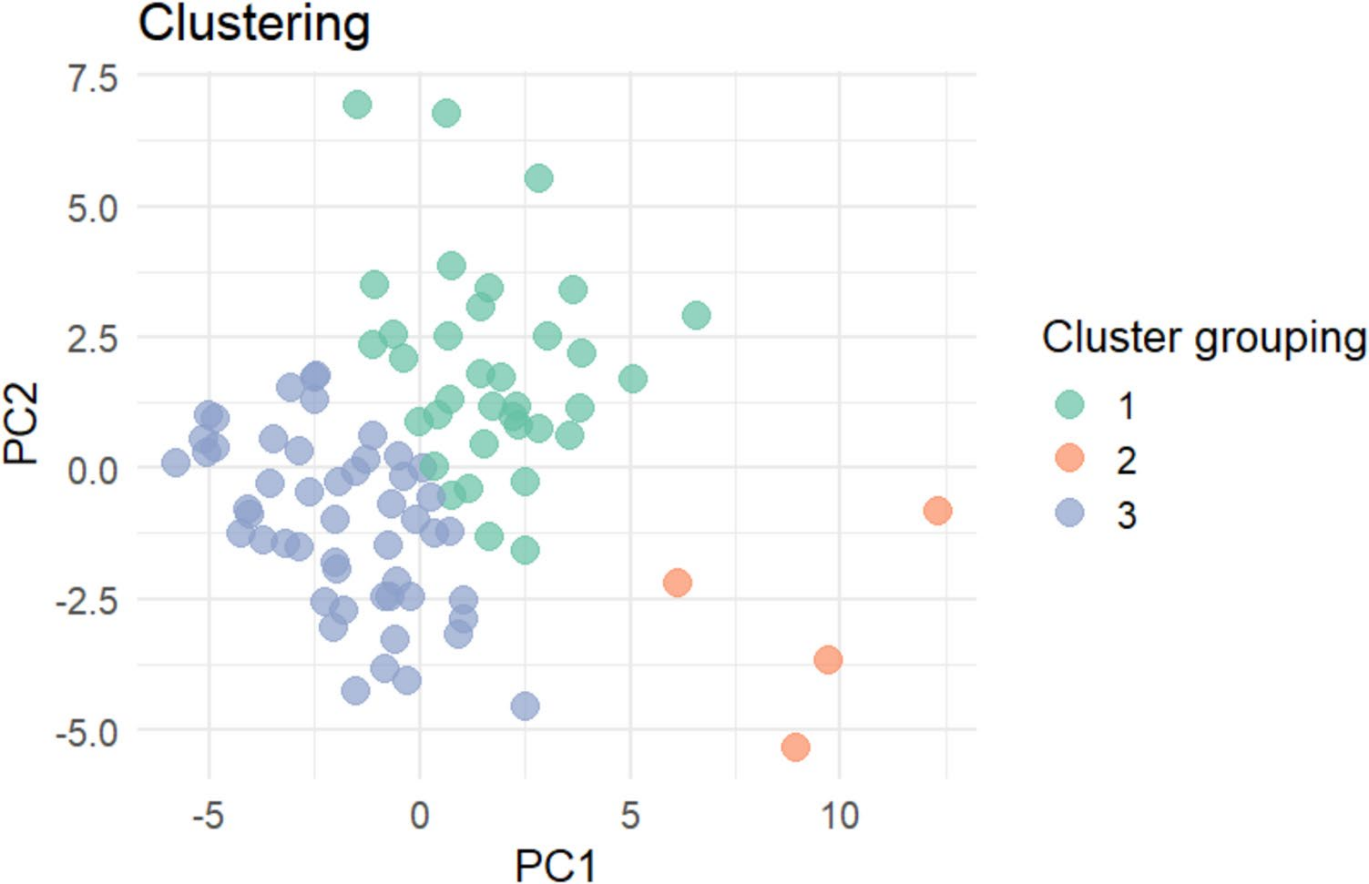
Cluster Grouping: This represents the group that each sample belongs to, based on similarities in their metabolite profiles, identified through K-means clustering

**Fig. 3:**
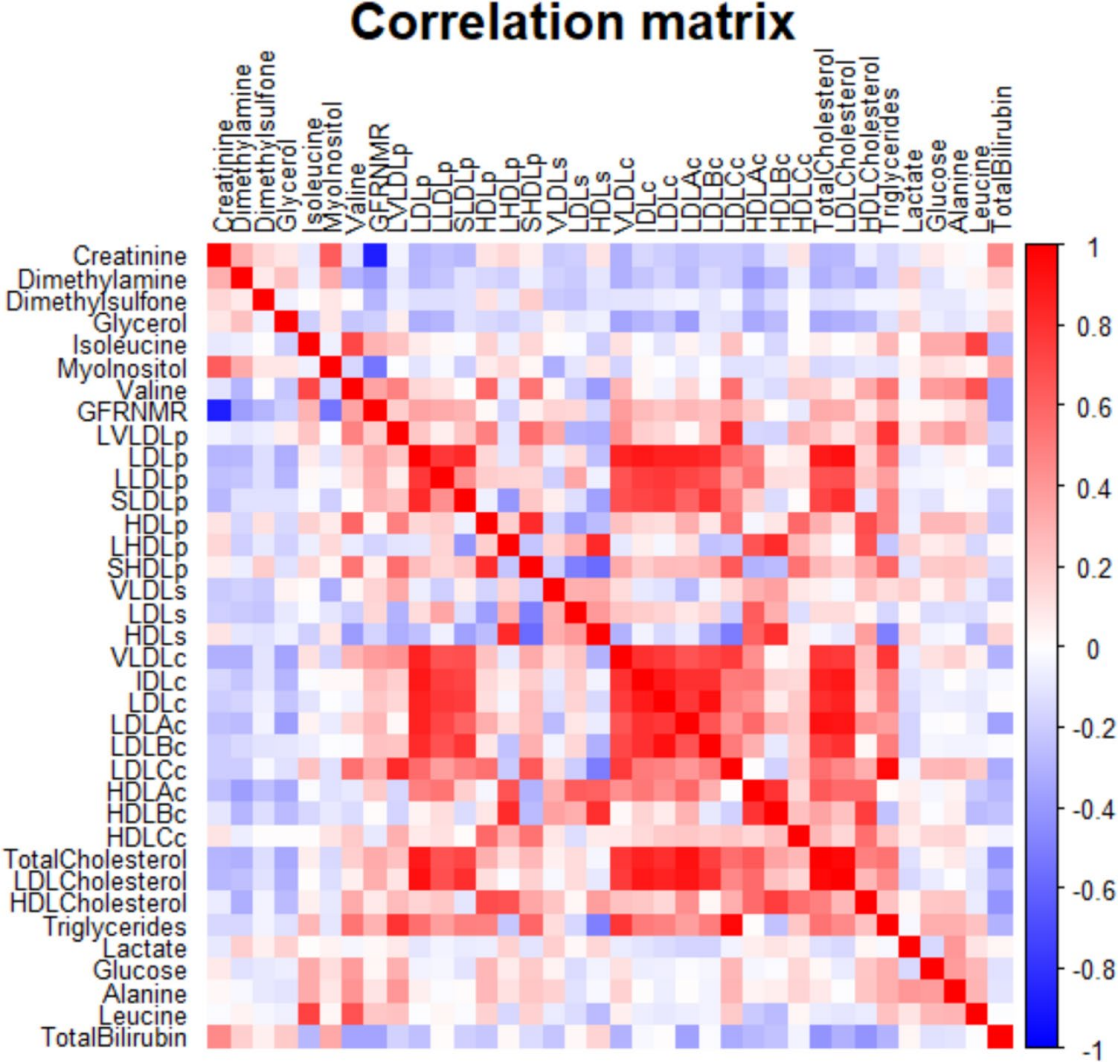
Heatmap of the correlation matrix between metabolites. The matrix was calculated using metabolite data, and the heatmap visualizes the relationships between them, with colors indicating the strength and direction of the correlations. Dark blue indicates a negative correlation between the two variables, where an increase in one variable corresponds to a decrease in the other. White indicates a null or very weak correlation, meaning the variables are either not correlated or only minimally correlated. Red indicates a positive correlation between the two variables, where an increase in one variable corresponds to an increase in the other. The darker the red, the stronger the positive correlation

Principal Component Analysis (PCA), clustering analysis, and heatmap visualization illustrate the distribution of the 39 analyzed proteins (Figs. 1, 2, 3).

### Lipids’ profile in HCC vs cirrhosis

The NMR serum profiles were compared between patients with HCC and patients with LC. A significant increase in the levels of cholesterol, some LDL subclasses, some HDL subclasses, Isoleucine, Valine, Triglycerides, Lactate, Alanine, Albumin and alpha Fetoprotein (AFP) was found in HCC with respect to LC (all *p* < 0.05). On the contrary, the levels of HDL_s, Dimethylamine, Glycerol and total Bilirubin were significantly lower in HCC than in LC (all *p* < 0.05). No significant differences between HCC and LC were detected for Creatine, Creatinine, Dimethysulfone, Myo-Inositol, some LDL and HDL subclasses, Glucose and Leucine. The analysis was supported by adjusted FDR p-values, ensuring robustness against multiple comparisons. The serum distribution of the complete NMR panel in the patients’ groups with LC with and without HCC is shown in Table 3.

**Table 3:** Metabolite profiles in HCC and liver cirrhosis patients

	HCC (early + adv) n = 60	LC n = 30	*p*-value	Adjusted FDR* p-values*
Creatine (µmol/L)	32.6 (24.6)	25.8 (13.2)	0.09	0.10
Creatinine (µmol/L)	95.5 (30.2)	111.2 (51.2)	0.12	0.13
Dimethylamine (µmol/L)	4.3 (0.6)	4.9 (1.3)	**0.02**	**0.03**
Dimethylsulfone (µmol/L)	12.2 (5.7)	10.7 (3.7)	0.14	0.14
Glycerol (µmol/L)	178.1 (62.0)	210.2 (80.7)	**0.04**	**0.04**
Isoleucine (µmol/L)	84.3 (19.2)	74.0 (17.3)	**0.01**	**0.02**
Myo-Inositol (µmol/L)	70.2 (23.7)	79.3 (50.4)	0.35	0.35
Valine (µmol/L)	284.7 (63.9)	216.2 (68.1)	** < 0.01**	** < 0.01**
LVLDL_p (nmol/L)	3.4 (2.6)	1.9 (0.8)	** < 0.01**	** < 0.01**
LDL_p (nmol/L)	1210.3 (535.3)	877.7 (435.2)	** < 0.01**	** < 0.01**
LLDL_p (nmol/L)	672.6 (261.6)	577.0 (284.0)	0.14	0.14
SLDL_p (nmol/L)	543.5 (335.8)	419.1 (239.3)	0.09	0.09
HDL_p (nmol/L)	21047.3 (8292.6)	13855.3 (9722.5)	** < 0.01**	** < 0.01**
LHDL_p (nmol/L)	5597.8 (2541.7)	6301.5 (3542.3)	0.36	0.37
SHDL_p (nmol/L)	16650.4 (7785.6)	11192.0 (6062.1)	** < 0.01**	** < 0.01**
VLDL_s (nm)	50.1 (4.2)	50.4 (4.4)	0.76	0.76
LDL_s (nm)	21.4 (0.5)	21.4 (0.4)	0.52	0.52
HDL_s (nm)	9.3 (0.5)	9.6 (0.4)	** < 0.01**	** < 0.01**
VLDL_c (mg/dL)	24.7 (8.9)	17.9 (8.1)	** < 0.01**	** < 0.01**
IDL_c (mg/dL)	45.8 (16.0)	41.0 (18.3)	0.20	0.21
LDL_c (mg/dL)	103.8 (37.4)	91.2 (36.2)	0.17	0.17
LDL_A_c (mg/dL)	35.0 (10.6)	27.5 (13.1)	** < 0.01**	** < 0.01**
LDL_B_c (mg/dL)	17.7 (11.6)	13.2 (7.7)	**0.04**	**0.05**
LDL_C_c (mg/dL)	4.1 (1.9)	2.4 (1.2)	** < 0.01**	** < 0.01**
HDL_A_c (mg/dL)	18.0 (5.3)	17.3 (7.2)	0.59	0.59
HDL_B_c (mg/dL)	15.3 (2.0)	15.8 (2.8)	0.43	0.43
HDL_C_c (mg/dL)	9.9 (6.3)	9.2 (5.2)	0.65	0.66
Total_Cholesterol (mg/dL)	169.8 (51.4)	133.8 (65.0)	** < 0.01**	**0.01**
LDL_Cholesterol (mg/dL)	105.5 (46.6)	83.2 (49.2)	**0.03**	**0.04**
HDL_Cholesterol (mg/dL)	42.1 (11.0)	36.4 (16.7)	0.10	0.10
Triglycerides (mg/dL)	120.8 (53.5)	74.7 (29.6)	** < 0.01**	** < 0.01**
Lactate (µmol/L)	30.4 (13.4)	29.3 (12.2)	0.71	0.71
Glucose (µmol/L)	131.2 (66.8)	113.2 (43.3)	0.12	0.13
Alanine (µmol/L)	490.4 (104.0)	429.0 (132.9)	**0.01**	**0.02**
Leucine (µmol/L)	154.7 (40.2)	141.8 (31.1)	0.19	0.20
Albumin (g/dL)	38.9 (5.7)	34.6 (7.3)	** < 0.01**	** < 0.01**
Total Bilirubin (micromol/L)	16.1 (7.8)	83.2 (120.5)	** < 0.01**	** < 0.01**
Alfa-Fetoprotein (ng/dL)	71,210.0 (522,061.3)	4.6 (7.4)	0.30	0.30

Serum concentrations were analyzed using the standardized AXINON platform, except for albumin, total bilirubin, and alpha-fetoprotein. Mean values and standard deviations (in brackets), as well as p-values and adjusted FDR p-values, are displayed

### Lipids’ profile in early HCC vs Cirrhosis and in advanced HCC vs Cirrhosis

Next, we compared the NMR profiles between HCC and LC, distinguishing between early and advanced HCC. In early HCC we found significantly higher levels of Valine, some LDL subclasses, some HDL subclasses, Triglycerides, Alanine, Albumin, and AFP, while we observed significantly lower levels of Dimethylamine and Bilirubin compared to LC (all *p* < 0.05) (Table 4). The same analysis applied to advanced HCC showed significant higher levels of Cholesterol concentration, some LDL subclasses, some HDL subclasses, Isoleucine, and Valine compared to LC, (all *p* < 0.05). No additional significant differences were found (Table 4). The findings were statistically validated by adjusted FDR p-values, accounting for multiple testing correction.

**Table 4:** NMR profiles in early HCC, advanced HCC and liver cirrhosis patients

	**HCC**			***p*-value**		***Adjusted***	***p-value***
	**Early** **n = 30**	**Advanced** **n = 30**	**LC** **n = 30**	**Early vs LC**	**Advanced vs LC**	**Early vs** **LC**	**Advanced vs LC**
Creatine (µmol/L)	32.2 (24.4)	33.0 (25.2)	25.8 (13.2)	0.22	0.18	0.22	0.19
Creatinine (µmol/L)	95.8 (28.6)	95.1 (32.4)	111.2 (51.2)	0.15	0.15	0.16	0.16
Dimethylamine (µmol/L)	4.3 (0.6)	4.4 (0.7)	4.9 (1.3)	**0.01**	0.06	**0.02**	0.06
Dimethylsulfone (µmol/L)	12.2 (5.1)	12.1 (6.4)	10.7 (3.7)	0.17	0.30	0.18	0.31
Glycerol (µmol/L)	173.0 (65.4)	183.6 (58.8)	210.2 (80.7)	**0.05**	0.15	**0.05**	0.16
Isoleucine (µmol/L)	83.2 (21.5)	85.5 (16.7)	74.0 (17.3)	0.07	**0.01**	0.07	**0.01**
Myo-Inositol (µmol/L)	71.3 (28.8)	69.1 (17.3)	79.3 (50.4)	0.45	0.30	0.45	0.30
Valine (µmol/L)	282.5 (72.6)	287.0 (54.4)	216.2 (68.1)	** < 0.01**	** < 0.01**	** < 0.01**	** < 0.01**
LVLDL_p (nmol/L)	3.7 (2.9)	3.0 (2.2)	1.9 (0.8)	** < 0.01**	**0.01**	** < 0.01**	**0.01**
LDL_p (nmol/L)	1132.1 (477.7)	1291.1 (586.4)	877.7 (435.2)	** < 0.01**	** < 0.01**	**0.05**	** < 0.01**
LLDL_p (nmol/L)	632.8 (215.0)	713.8 (300.7)	577.0 (284.0)	0.41	0.09	0.42	0.10
SLDL_p (nmol/L)	506.4 (343.5)	581.9 (329.2)	419.1 (239.3)	0.28	**0.04**	0.28	**0.04**
HDL_p (nmol/L)	20798.6 (7999.7)	21304.6 (8719.5)	13855.3 (9722.5)	** < 0.01**	** < 0.01**	** < 0.01**	** < 0.01**
LHDL_p (nmol/L)	6039.9 (2806.9)	5140.3 (2189.9)	6301.5 (3542.3)	0.75	0.15	0.76	0.16
SHDL_p (nmol/L)	15910.8 (7652.0)	17444.8 (7993.8)	11192.0 (6062.1)	**0.01**	** < 0.01**	**0.04**	** < 0.01**
VLDL_s (nm)	50.5 (4.8)	49.7 (3.5)	50.4 (4.4)	0.91	0.49	0.92	0.49
LDL_s (nm)	21.3 (0.4)	21.5 (0.5)	21.4 (0.4)	0.16	0.77	0.17	0.78
HDL_s (nm)	9.3 (0.5)	9.2 (0.4)	9.6 (0.4)	0.06	** < 0.01**	0.07	** < 0.01**
VLDL_c (mg/dL)	24.2 (7.4)	25.2 (10.4)	17.9 (8.1)	** < 0.01**	** < 0.01**	** < 0.01**	** < 0.01**
IDL_c (mg/dL)	43.8 (14.6)	47.9 (17.4)	41.0 (18.3)	0.52	0.14	0.52	0.14
LDL_c (mg/dL)	97.3 (35.3)	109.8 (38.8)	91.2 (36.2)	0.54	0.08	0.55	0.08
LDL_A_c (mg/dL)	32.3 (10.9)	37.9 (9.7)	27.5 (13.1)	0.12	** < 0.01**	0.13	** < 0.01**
LDL_B_c (mg/dL)	14.9 (10.5)	20.4 (12.2)	13.2 (7.7)	0.52	**0.01**	0.53	**0.01**
LDL_C_c (mg/dL)	4.1 -2.1)	4.0 (1.8)	2.4 (1.2)	** < 0.01**	** < 0.01**	** < 0.01**	** < 0.01**
HDL_A_c (mg/dL)	17.5 (6.1)	18.5 (4.4)	17.3 (7.2)	0.87	0.43	0.88	0.44
HDL_B_c (mg/dL)	15.4 (2.3)	15.2 (1.8)	15.8 (2.8)	0.60	0.35	0.60	0.35
HDL_C_c (mg/dL)	9.2 (5.9)	10.6 (6.7)	9.2 (5.2)	0.97	0.45	0.98	0.46
Total_Cholesterol (mg/dL)	159.0 (53.8)	181.1 (47.2)	133.8 (65.0)	0.10	** < 0.01**	0.11	** < 0.01**
LDL_Cholesterol (mg/dL)	95.1 (47.1)	116.2 (44.3)	83.2 (49.2)	0.34	** < 0.01**	0.34	**0.01**
HDL_Cholesterol (mg/dL)	42.1 (11.5)	42.0 (10.8)	36.4 (16.7)	0.13	0.13	0.13	0.13
Triglycerides (mg/dL)	124.4 (53.0)	117.2 (54.6)	74.7 (29.6)	** < 0.01**	** < 0.01**	** < 0.01**	** < 0.01**
Lactate (µmol/L)	36.9 (15.4)	24.2 (7.2)	29.3 (12.2)	0.05	0.07	0.06	0.07
Glucose (µmol/L)	130.7 (83.0)	131.7 (45.8)	113.2 (43.3)	0.31	0.11	0.31	0.12
Alanine (µmol/L)	508.4 (112.1)	471.8 (93.1)	429.0 (132.9)	**0.01**	0.15	**0.02**	0.16
Leucine (µmol/L)	158.4 (44.0)	150.7 (36.2)	141.8 (31.1)	0.15	0.38	0.15	0.38
Albumin (g/dL)	39.1 (5.3)	38.7 (6.1)	34.6 (7.3)	** < 0.01**	**0.02**	**0.01**	**0.02**
Total Bilirubin (micromol/L)	18,3 (8,1)	13,8 (7,0)	83,2 (120,5)	** < 0.01**	** < 0.01**	**0.01**	** < 0.01**
Alfa-Fetoprotein (ng/dL)	137,250.8 (738,681.1)	5169.2 (12,079.8)	4.6 (7.4)	0.32	**0.02**	0.33	**0.03**

Serum concentrations were analysed using the standardized AXINON platform. The mean values and standard deviation (in brackets) as well as p values and adjusted FDR p values, are displayed

### Liver insufficiency

We further investigated NMR profiles for different degrees of liver failure. In patients with early HCC and CPS A we observed a higher concentration of Creatinine, Dimethylamine, Valine, some HDL subclasses, Albumin and a lower concentration of LDL_s, HDL_A_c and total Bilirubin than in patients with early HCC and CPS-B/C (all *p* < 0.05) (Table 5A). Furtermore, in patients with advanced HCC and CPS A we observed a higher concentration of Dimethylamine, some HDL subclasses, LDL_s, Albumin and a lower concentration of some HDL subclasses and LDL subclasse than in patients with advanced HCC and CPS B/C (all *p* < 0.05) (Table 5B). Finally, cirrhotic patients with CPS A presented a higher level of Valine, some HDL subclasses, total Cholesterol, Alanine and Albumin and a lower level of Creatine, Dimethylsulfone and total Bilirubin when compared to patients with CPS B/C (all *p* < 0.05) (Table 5C).

We conducted a logistic regression analysis to assess the relationship between various metabolites and the presence of HCC, adjusting for liver function (Child–Pugh score) and tumor stage. Several metabolites showed significant associations with HCC presence. Specifically, Dymethilsulphone (p = 0.02), Valine (p = 0.04), LDL_p (p = 0.04), HDL_s (p = 0.01), HDL_B_c (p = 0.01), Triglycerides (p = 0.02), Alanine (p = 0.05), and Total Bilirubin (p < 0.01) were significantly associated with HCC. These findings suggest that alterations in these metabolites are independently linked to the presence of HCC, even after adjusting for liver function and tumor stage. This highlights their potential as biomarkers for HCC, independent of liver function and disease progression.

Additionally, to explore the independent effects of albumin and total bilirubin on HCC stage while controlling for liver function, we performed a linear regression analysis. Total bilirubin showed significant correlations with both HCC stage/cirrhosis (p < 0.01) and the Child–Pugh score (p < 0.01). Albumin was significantly associated with the Child–Pugh score (p < 0.01) and showed a notable interaction between HCC stage/cirrhosis and the Child–Pugh score (p < 0.01). However, the interaction between bilirubin and the combined effects of HCC stage/cirrhosis and Child–Pugh was not significant (p > 0.05). These results suggest that while albumin is influenced by both liver function and HCC stage, bilirubin remains independently associated with these factors.

### MRI follow up of liver cirrhosis

**Fig. 4:**
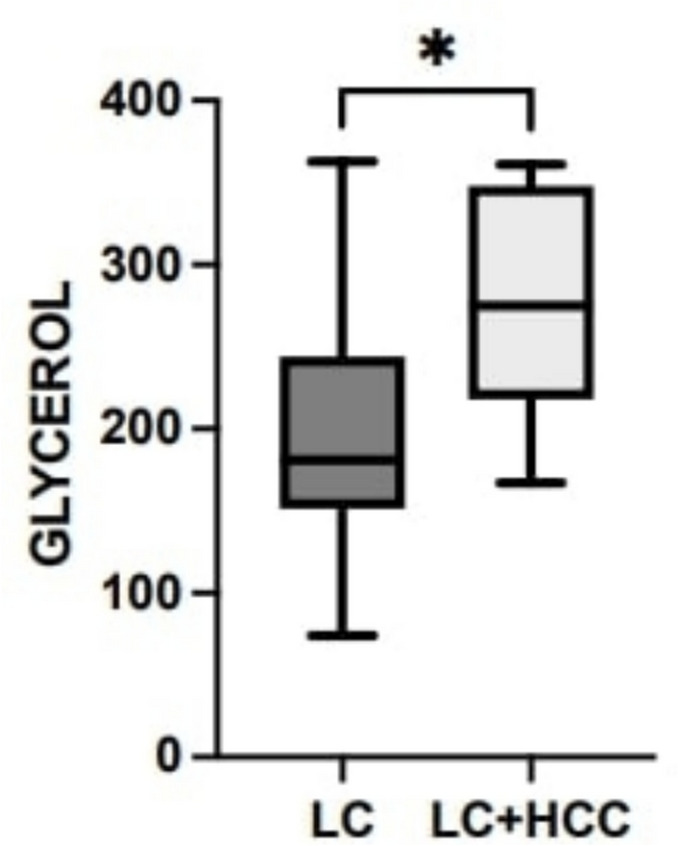
Boxplot showing difference in the glycerol levels between cirrhotic patients who developed HCC and patients who did not. Comparison was performed using the Mann–Whitney *U* test and the corresponding p value of significant difference is indicated in the graphs (*p ≤ 0.05)

In order to evaluate if NMR profiles might have a predictive value in HCC development, we evaluated the clinical history of the patients from the LC group at later follow ups. We found that 16% (n = 5) of the patients developed HCC after the conclusion of the study. Interestingly, the NMR profiles in these patients showed a significant difference in the gylcerol levels between cirrhotic patients who developed HCC and patients who did not (*p* < 0.05) (Fig. 4). In addition, in patients who developed HCC we observed a decreasing trend of Dimethylamine, aligning them more to the HCC group than to the LC group (data not shown).

**Fig. 5:**
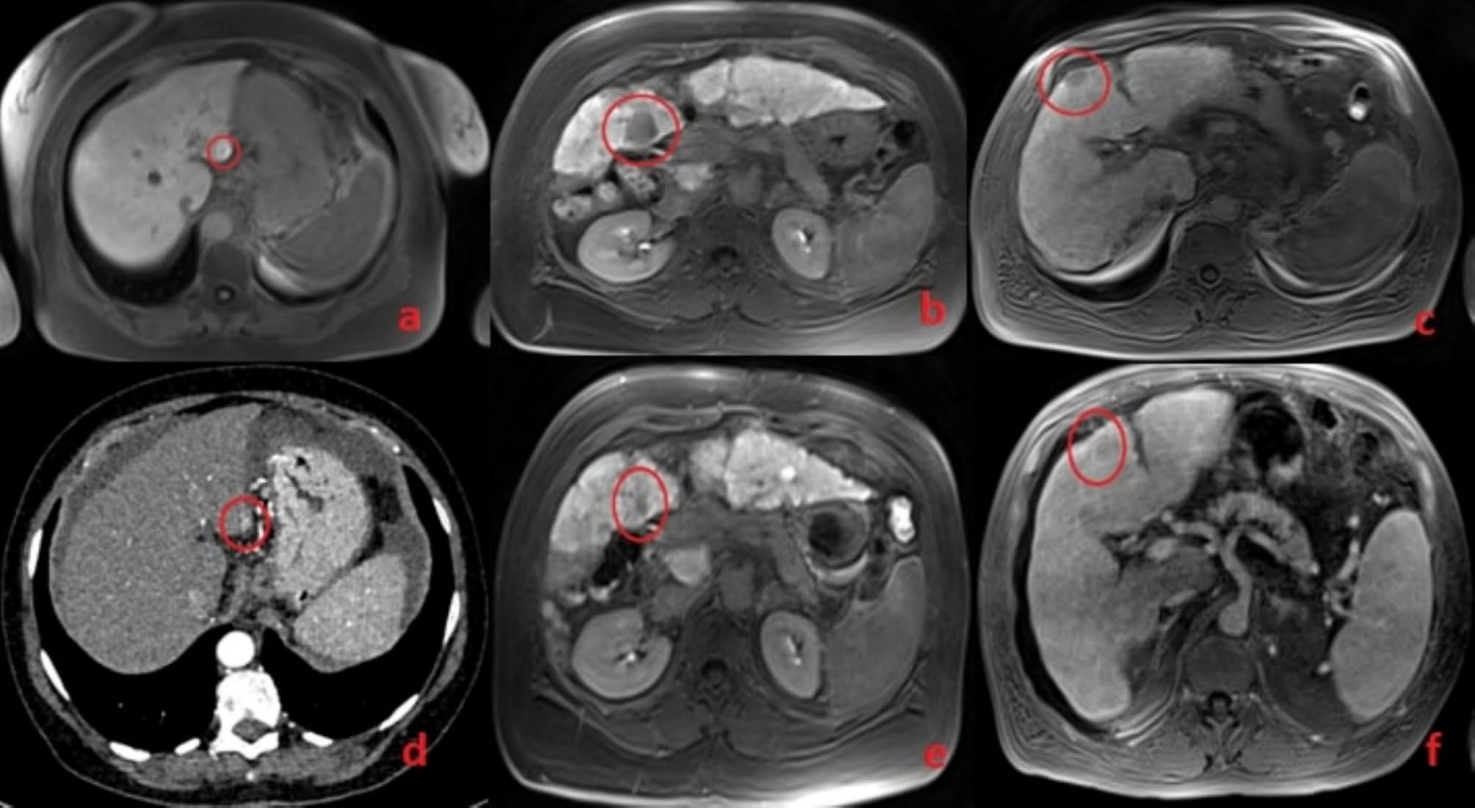
MRI and CT scans of LC patients at the time of NMR profiling (Images **d**, **e**, and **f**) and at the time of diagnosis (Images **a**, **b**, and **c**). The lower images (**d**, **e**, **f**) show potential precursor lesions that later developed into HCC at baseline, while the upper images (**a** and **c**) display the lesions identified as HCC during the 6-month follow-up. Image b shows the lesion identified at the 1-year follow-up

In patients who developed the tumor, imaging follow-ups were performed every 4–6 months. From a retrospective analysis of the scans obtained at the time of the NMR profiling, we observed that precursor lesions were already present in three out of five patients. However, based on size and hemodynamic features, those lesions were not classified as overt HCC (Fig. 5). Specifically, HCC was diagnosed at 6 months in 2 patients, at 1 year in one patient, and at 2 years in the remaining two patients from the time of sampling.

## Discussion

From the comparison of the NMR profiles, we observed that HCC patients have significantly higher levels of cholesterol, some LDL subclasses, some HDL subclasses, Isoleucine, Valine, Triglycerides, Lactate, Alanine, Albumin and AFP, and significant lower levels of HDL_s, Dimethylamine, Glycerol and total Bilirubin. Among the different lipids, we find notable changes in Dimethylamine and Glycerol in the patients’ groups.

A main source of dimethylamine is the hydroxylation of asymmetric dimethylarginine (ADMA), an endogenous inhibitor of nitric oxide synthase, closely linked to LC and with oncogenic potential (Barnes and Magee 1954; George et al. 2001; Haggerty and Holsapple 1990). We found in our cohort decreased levels of Dimethylamine in patients with HCC vs patients with LC without HCC. Thus, we hypothesize that lower levels of Dimethylamine could be associated with higher levels of ADMA, indicating a poorer liver function and an increased risk of developing HCC. Glycerol levels in patients with LC were significantly higher than patients with HCC. This data confirms previous results showing a decrease of glycerol phosphate and other energy metabolite concentrations in patients with HCC (Beyoğlu et al. 2013). Furthermore, a recent study using glycerol to treat pre-neoplastic liver lesions showed a decrease in the volume of the preneoplastic lesion by a down regulation of the proliferative status of liver foci (Capiglioni et al. 2018). The neoplastic lesions can also reprogram its metabolism to gain a survival advantage as reported by Warburg (Kim et al. 2009).

The central role of the liver in the metabolism of carbohydrates, amino acids, and lipids supports the results of this study. Metabolic remodelling can lead to increased levels of amino acids and lactate production, as demonstrated in several studies (Beyoğlu et al. 2013; Kim et al. 2009; Morine et al. 2022). In our study, we observed significantly higher levels of amino acids (Alanine, Leucine, Isoleucine) and lactate in both early and advanced HCC compared to patients with LC.

Contrasting results about triglycerides values in HCC are found in the literature (Motta et al. 2001; Alsabti 1979; Ooi et al. 2005; Jiang et al. 2006). In a study involving 40 HCC patients and 25 healthy patient controls, Motta et al. observed lower levels of triglycerides in HCC patients compared to controls (Motta et al. 2001). Alsabti et al. (1979) found that serum triglycerides levels were elevated in HCC patients compared to individuals with LC. Ooi et al. (2005) reported no significant difference in plasma triglycerides levels between HCC patients and controls. In our cohort, a significant difference between patients with LC and HCC was noted, with increased value in patients with HCC. Triglycerides might be decreased in HCC due to impaired lipogenesis, increased lipolysis, and the nutritional status of the patients that lead to malabsorption. On the other hand, triglycerides might be increased in HCC due to insulin resistance, dysfunctional lipid metabolism, altered lipoprotein production, and chronic inflammation. We hypothesized that the increased level of triglycerides was caused by an impairment of the liver’s ability to metabolize and regulate lipids.

In a study involving approximately 9000 patients, Li et al. demonstrated a significant negative correlation between serum cholesterol levels and HCC (Li 1993). This correlation was also present in chronic hepatitis and LC. The lower levels of cholesterol may be attributed to increased consumption by tumor cells (Eggens et al. 1990). In cases of HCC and chronic liver diseases, there is impairment in the synthesis and metabolism of cholesterol (Jiang et al. 2006 Mar), which can result in a reduction of plasma cholesterol levels. The elevated cholesterol levels could be attributed to altered metabolism or an increased metabolic demand for growth and proliferation, resulting in a rise in circulating levels (Zhou and Sun 2021; Suzuki et al. 2021; Athavale et al. 2018). Furthermore, in HCC, compromised liver function may lead to lipid accumulation in the blood, including cholesterol. In our cohort cholesterol levels were higher in patients with HCC. Following the levels of triglycerides, our cohort presented an increased value of VLDL and LDL fractions among patients with LC and HCC. This result contradicts Motta et al., (2001) who demonstrated a significant correlation between a decrease in LDL-C levels and the severity of chronic liver disease. There was a significant correlation in the HDL fraction in our cohort, showing an increased amount of HDL_s in patients with LC compared to patients with HCC, consistent with existing literature (Jiang et al. 2006; Ahaneku et al. 1992; Trieb et al. 2016). HDL_s stand for the size of HDL particles suggesting larger particles in LC than in HCC. We hypothesize that the increasing value of HDL is due to its role in modulating inflammatory responses and a potential increase in cholesterol metabolism. However, there was a decreasing trend of HDL_p and SHDL_p, in disagreement with the literature. HDL_p corresponds to the number of HDL particles, on the other hand HDL_c means the concentration of cholestrol in HDL particles.

Furthermore, there were 62 patients with a CPS A and 28 with a CPS B or C. Looking closer, 24 patients were classified with a CPS A and 6 with a CPS B/C in advanced HCC group; 25 patients were classified with a CPS A and 5 with a CPS B/C in early HCC group; 13 patients were classified with a CPS A and 17 with a CPS B/C in LC without HCC group.

The analysis within patients with cirrhosis without HCC revealed significant differences between patients in the CPS A vs B/C groups regarding Creatine, Dimethysulfone, Valine, HDL_p, HDL_A_c, HDL_B_c, HDL_C_c, Total Cholesterol, Alanine, Albumin, and Total Bilirubin.

On the other hand, in patients with HCC and CPS A vs B/C, significant differences were observed in Creatinine, Dimethylamine, Valine, HDL_p, SHDL_p, LDL_s, HDL_A_c, HDL_C_c, Albumin, and Total Bilirubin.

Valine, HDL_p, Albumin and Total Bilirubin were found to be significantly different both in patients with cirrhosis vs HCC and in patients with HCC and cirrhosis with CPS A vs B/C. Thus, we speculated that only 4 markers out of 20 (20%) appeared to be influenced by liver failure.

The higher distribution in our cohort of patients with a CPS B/C vs A in LC patients without HCC (17vs13) allow us to exclude possible markers indicative of liver failure. Furthermore, the higher distribution of patients with CPS A vs B/C in HCC groups (49vs11) permit us to further select only expression markers of HCC transformation.

Finally, we have investigated the history of the control patients with LC without HCC at the time of the study and found that five patients had developed HCC. We found that these patients had a decreased value of Dimethylamine, aligning more closely to the patients with early HCC rather than to their belonging group. We hypothesize that only five patients had developed HCC because most of our control group had either done a liver transplant shortly after our blood sample (about nine patients) or died from other causes. Furthermore, we speculate that having a retrospective revision of the images in both patients with decreased Dimethylamine, could reveal some ancestral sign of HCC at the time of the NMR profiling. We suggest that more frequent and strict monitoring be implemented for patients with decreased levels of Dimethylamine.

The strength of our study lies in the use of NMR and the comparable degree of liver failure within the cohort.

NMR spectroscopy allows reliable, fast, and flexible metabolome analysis and, when combined with machine learning and artificial intelligence methods, enables discovery of diagnostic multi-marker combinations that can overcome the limitations of single biomarkers. NMR provides high informative content with detection of up to 400 metabolites in a single analytical step, including advanced lipoprotein analysis. While sensitivity is lower compared to other technologies, strengths of NMR include its high analytical reproducibility, the simplicity of sample preparation, and the physical measurement allow sample analysis without altering the sample ‘s composition. The NMR system used in this study is designed for integration into standard laboratories, high throughput analyses and fully automated data processing, obviating the need for specially trained NMR experts. These are prerequisites for cost-effective implementation of metabolomic-based diagnostics into clinical routine, compensating for investment costs of an NMR device. The small sample size and the heterogeneity of the cohort does not permit further metabolomic analyzation and deeper correlation with clinical data.

Limitations of the present study include the small sample size with few metabolic HCC cases and the absence of follow-up controls, especially in patients with LC. Further studies with larger cohorts are necessary. Additionally, since the samples were collected over an extended period, potential batch effects may be present but have not been examined. Without external validation, the conclusions may slightly overstate the predictive potential, and this limitation should be explicitly considered. Furthermore, without functional validation, the biological role of dimethylamine and glycerol in HCC progression remains unclear, as these metabolites could be byproducts of metabolic dysfunction rather than direct contributors to tumor biology.

## Conclusion

The current study revealed that an NMR-based assessment of lipidomic and metabolomic profiles has the potential to identify individual metabolic biomarker candidates capable of predicting early HCC. Due to its reliable, easy, and reproducible characterization of lipidomic and metabolomic profiles, NMR-based metabolomic analysis emerges as a promising tool for developing novel biomarkers for the diagnostic and therapeutic management of HCC patients. Subsequent studies with larger sample sizes would allow exploiting the full potential of this metabolomic approach by enabling multivariate analysis and machine learning to identify multi-marker combinations for precision diagnostics. Furthermore, we focused our attention on Dimethylamine, hypothesizing that a more strict control should be done on patients where this marker is decreased.

## Supplementary Information

Below is the link to the electronic supplementary material.

Supplementary file1 (DOCX 36 KB)

## Data Availability

All data being analyzed as part of this study are included in this manuscript and the supplementary materials. Further inquiries can be sent to the corresponding author.

## Abbreviations

AFP: Alpha-fetoprotein
BCLC: Barcelona clinic liver cancer
CPS: Child-Pugh score
HCC: Hepatocellular carcinoma
LC: Liver cirrhosis
NMR: Nuclear magnetic resonance
NAFLD: Nonalcoholic fatty liver diseas
MAFLD: Metabolic dysfunction-associated fatty liver disease
