# Supplementary material for: Nuclear magnetic resonance-based lipid metabolite profiles for differentiation of patients with liver cirrhosis with and without hepatocellular carcinoma
Source: J Cancer Res Clin Oncol. 2025 Apr 4;151(4):131. doi: 10.1007/s00432-025-06178-x (PMC11971233; doi:10.1007/s00432-025-06178-x)
Supplement: Supplementary file 1 — Supplementary file1 (DOCX 36 KB) [file 432_2025_6178_MOESM1_ESM.docx]

**Supplementary tables**

**Table 5A. NMR profiles in early HCC with Child-Pugh score A and Child-Pugh score B/C.** Serum concentrations were analysed using the standardized AXINON platform. The mean values and standard deviation (in brackets) as well as the *p*-values are displayed.

|  | **Early HCC + CPS-A**  **n=25** | **Early HCC + CPS-B/C**  **n=5** | ***p*-value** |
| --- | --- | --- | --- |
| Creatine | 32.8 (25.8) | 29.0 (18.3) | 0.7557 |
| Creatinine | 100.9 (23.6) | 71.0 (39.9) | **0.0303** |
| Dimethylamine | 4.3 (0.6) | 4.2 (0.4) | 0.785 |
| Dimethylsulfone | 12.1 (4.9) | 12.8 (6.5) | 0.7893 |
| Glycerol | 173.4 (69.7) | 171.0 (42.2) | 0.9418 |
| Isoleucine | 84.0 (22.1) | 79.2 (20.1) | 0.6571 |
| Myo_Inositol | 68.7 (16.6) | 84.0 (64.1) | 0.6237 |
| Valine | 292.4 (70.6) | 233.2 (68.2) | 0.0967 |
| LVLDL_p | 3.8 (3.1) | 3.4 (2.0) | 0.7707 |
| LDL_p | 1111.3 (390.0) | 1236.2 (851.8) | 0.7629 |
| LLDL_p | 649.6 (221.1) | 549.0 (176.6) | 0.3486 |
| SLDL_p | 470.2 (237.1) | 687.2 (684.8) | 0.5207 |
| HDL_p | 22592.7 (7113.7) | 11828.0 (6294.9) | **0.004** |
| LHDL_p | 6222.0 (2961.7) | 5129.6 (1801.7) | 0.4366 |
| SHDL_p | 16956.2 (7528.1) | 9377.0 (5205.4) | 0.0647 |
| VLDL_s | 50.2 (4.8) | 51.8 (5.0) | 0.5118 |
| LDL_s | 21.3 (0.4) | 21.4 (0.5) | 0.5949 |
| HDL_s | 9.3 (0.6) | 9.5 (0.4) | 0.3366 |
| VLDL_c | 24.0 (6.5) | 25.2 (11.7) | 0.752 |
| IDL_c | 42.6 (9.5) | 49.6 (30.7) | 0.6382 |
| LDL_c | 94.8 (25.8) | 111.9 (75.0) | 0.6804 |
| LDL_A_c | 32.1 (9.5) | 33.7 (17.9) | 0.8549 |
| LDL_B_c | 14.2 (8.3) | 18.7 (20.6) | 0.699 |
| LDL_C_c | 4.2 (2.2) | 3.6 (1.7) | 0.5163 |
| HDL_A_c | 17.3 (5.9) | 18.7 (7.8) | 0.6583 |
| HDL_B_c | 15.4 (2.3) | 15.7 (2.5) | 0.7547 |
| HDL_C_c | 9.2 (5.5) | 9.4 (10.2) | 0.9527 |
| Total_Cholesterol | 156.4 (37.3) | 172.2 (111.3) | 0.768 |
| LDL_Cholesterol | 90.7 (31.0) | 116.8 (97.9) | 0.5859 |
| HDL_Cholesterol | 43.4 (11.4) | 35.4 (10.0) | 0.1574 |
| Triglycerides | 127.5 (54.1) | 108.8 (49.3) | 0.4816 |
| Lactate | 38.2 (16.0) | 30.9 (12.4) | 0.4028 |
| Glucose | 132.8 (86.8) | 120.0 (67.6) | 0.7583 |
| Alanine | 511.6 (113.9) | 492.6 (113.7) | 0.7364 |
| Leucine | 159.2 (47.4) | 154.0 (13.3) | 0.8325 |
| Albumin | 40.2 (4.6) | 33.4 (5.0) | **0.0056** |
| Total Bilirubin | 16.6 (6.9) | 26.8 (9.2) | **0.0085** |
| Alfa-Fetoprotein | 165843.5 (811986.0) | 5.9 (2.9) | 0.3275 |

**Table 5B. NMR profiles in advanced HCC with Child-Pugh score A and Child-Pugh score B/C.** Serum concentrations were analysed using the standardized AXINON platform. The mean values and standard deviation (in brackets) as well as the *p*-values are displayed.

|  | **Advanced HCC + CPS-A**  **n=24** | **Advanced HCC + CPS-B/C**  **n=6** | ***p*-value** |
| --- | --- | --- | --- |
| Creatine | 32.2 (23.3) | 35.8 (33.9) | 0.7629 |
| Creatinine | 100.5 (31.2) | 75.5 (31.6) | 0.0946 |
| Dimethylamine | 4.5 (0.7) | 4.0 (0.0) | **0.0046** |
| Dimethylsulfone | 12.0 (6.2) | 12.3 (7.7) | 0.9247 |
| Glycerol | 184.5 (58.0) | 180.5 (67.2) | 0.8858 |
| Isoleucine | 85.2 (17.9) | 86.5 (12.5) | 0.8721 |
| Myo-Inositol | 70.2 (18.3) | 65.0 (13.5) | 0.5219 |
| Valine | 295.5 (54.8) | 256.0 (43.2) | 0.1167 |
| LVLDL_p | 3.2 (2.3) | 2.4 (1.4) | 0.387 |
| LDL_p | 1230.9 (394.1) | 1522.0 (1077.8) | 0.5423 |
| LLDL_p | 691.1 (248.8) | 800.8 (471.8) | 0.6029 |
| SLDL_p | 545.6 (206.2) | 721.2 (624.9) | 0.526 |
| HDL_p | 23707.1 (7994.1) | 12094.7 (4030.5) | **0.002** |
| LHDL_p | 4945.0 (1925.5) | 5889.3 (3113.5) | 0.3562 |
| SHDL_p | 19039.6 (7472.9) | 8274.8 (3559.3) | **0.0099** |
| VLDL_s | 49.0 (3.5) | 52.4 (1.9) | **0.0306** |
| LDL_s | 21.3 (0.4) | 21.9 (0.4) | **0.0027** |
| HDL_s | 9.1 (0.3) | 9.5 (0.6) | 0.2327 |
| VLDL_c | 23.6 (5.9) | 31.4 (20.0) | 0.3862 |
| IDL_c | 45.5 (9.3) | 57.1 (34.6) | 0.4509 |
| LDL_c | 106.7 (24.8) | 121.8 (74.3) | 0.6414 |
| LDL_A_c | 37.9 (8.5) | 37.7 (14.5) | 0.9586 |
| LDL_B_c | 19.5 (8.3) | 23.9 (22.7) | 0.6543 |
| LDL_C_c | 4.0 (1.6) | 3.9 (2.5) | 0.9111 |
| HDL_A_c | 17.2 (3.5) | 23.4 (4.5) | **0.0012** |
| HDL_B_c | 14.9 (1.5) | 16.1 (2.6) | 0.1586 |
| HDL_C_c | 12.4 (6.5) | 4.2 (2.0) | **<.0001** |
| Total_Cholesterol | 177.3 (32.4) | 195.7 (86.7) | 0.6298 |
| LDL_Cholesterol | 111.6 (28.5) | 134.2 (83.2) | 0.5397 |
| HDL_Cholesterol | 43.3 (10.7) | 37.2 (10.9) | 0.2217 |
| Triglycerides | 117.4 (46.5) | 116.2 (84.8) | 0.973 |
| Lactate | 22.6 (5.2) | 29.9 (10.6) | 0.2057 |
| Glucose | 135.7 (49.1) | 116.7 (28.4) | 0.3753 |
| Alanine | 463.7 (96.9) | 502.8 (76.1) | 0.3692 |
| Leucine | 154.2 (39.0) | 135.8 (14.9) | 0.3168 |
| Albumin | 40.7 (4.8) | 30.3 (2.4) | **<.0001** |
| Total Bilirubin | 12.9 (6.8) | 17.3 (7.1) | 0.1643 |
| Alfa-Fetoprotein | 3584.3 (9620.2) | 11244.6 (18817.4) | 0.3738 |

**Table 5C. NMR profiles in liver cirrhotic patients with Child-Pugh score A and Child-Pugh score B/C.** Serum concentrations were analysed using the standardized AXINON platform. The mean values and standard deviation (in brackets) as well as the *p*-values are displayed

|  | **LC + CPS-A**  **n=13** | **LC + CPS- B/C**  **n=17** | ***p*-value** |
| --- | --- | --- | --- |
| Creatine | 19,1 (6,1) | 30,9 (14,9) | **0.0069** |
| Creatinine | 98,7 (28,7) | 120,8 (62,6) | 0.2103 |
| Dimethylamine | 4,7 (1,3) | 5,1 (1,2) | 0.4428 |
| Dimethylsulfone | 9,0 (1,8) | 11,9 (4,4) | **0.0196** |
| Glycerol | 207,5 (98,0) | 212,3 (67,8) | 0.8762 |
| Isoleucine | 80,5 (15,8) | 68,9 (17,1) | 0.068 |
| Myo_Inositol | 74,1 (24,1) | 83,4 (64,3) | 0.5899 |
| Valine | 251,3 (63,4) | 189,3 (60,2) | **0.0108** |
| LVLDL_p | 2,2 (1,1) | 1,7 (0,3) | 0.1728 |
| LDL_p | 954,7 (497,4) | 818,9 (386,3) | 0.4065 |
| LLDL_p | 659,5 (366,4) | 507,3 (176,6) | 0.2288 |
| SLDL_p | 383,8 (189,4) | 445,0 (273,7) | 0.5305 |
| HDL_p | 19532,6 (9009,6) | 8583,5 (7208,7) | **0.0018** |
| LHDL_p | 7513,8 (4068,9) | 5089,2 (2530,0) | 0.0805 |
| SHDL_p | 13038,5 (6260,3) | 8791,5 (5123,3) | 0.0964 |
| VLDL_s | 51,7 (4,2) | 49,4 (4,4) | 0.1597 |
| LDL_s | 21,5 (0,4) | 21,4 (0,4) | 0.654 |
| HDL_s | 9,6 (0,5) | 9,5 (0,4) | 0.3789 |
| VLDL_c | 20,2 (8,9) | 16,2 (7,2) | 0.1888 |
| IDL_c | 43,3 (23,2) | 39,2 (14,0) | 0.5503 |
| LDL_c | 91,9 (45,5) | 90,3 (21,1) | 0.9113 |
| LDL_A_c | 31,9 (14,6) | 24,2 (11,2) | 0.1121 |
| LDL_B_c | 12,9 (9,2) | 13,6 (5,6) | 0.8344 |
| LDL_C_c | 2,8 (1,4) | 2,1 (1,0) | 0.0872 |
| HDL_A_c | 20,3 (8,2) | 15,0 (5,5) | **0.0406** |
| HDL_B_c | 17,3 (2,9) | 14,6 (2,1) | **0.0075** |
| HDL_C_c | 11,2 (4,9) | 5,7 (3,9) | **0.0203** |
| Total_Cholesterol | 160,7 (67,1) | 113,2 (56,9) | **0.0454** |
| LDL_Cholesterol | 96,5 (54,4) | 73,0 (43,7) | 0.2007 |
| HDL_Cholesterol | 47,8 (16,4) | 27,8 (11,1) | **0.0004** |
| Triglycerides | 84,1 (32,5) | 67,5 (25,8) | 0.1313 |
| Lactate | 30,8 (16,1) | 28,0 (7,7) | 0.5768 |
| Glucose | 126,9 (58,3) | 102,6 (24,2) | 0.1784 |
| Alanine | 500,7 (144,4) | 374,2 (94,9) | **0.0073** |
| Leucine | 147,2 (28,1) | 133,6 (35,4) | 0.3532 |
| Albumin | 40,2 (5,2) | 30,3 (5,5) | **<,0001** |
| Total Bilirubin | 23,2 (17,0) | 129,1 (144,8) | **0.0084** |
| Alfa-Fetoprotein | 5,4 (10,4) | 4,1 (4,2) | 0.6764 |
